# Mediterranean Alcohol-Drinking Pattern and the Incidence of Cardiovascular Disease and Cardiovascular Mortality: The SUN Project

**DOI:** 10.3390/nu7115456

**Published:** 2015-11-05

**Authors:** Aitor Hernandez-Hernandez, Alfredo Gea, Miguel Ruiz-Canela, Estefania Toledo, Juan-José Beunza, Maira Bes-Rastrollo, Miguel A. Martinez-Gonzalez

**Affiliations:** 1 Department of Cardiology, Clínica Universidad de Navarra, 31009 Pamplona, Spain; aitorhernandez86@gmail.com; 2 Department of Preventive Medicine and Public Health, School of Medicine, University of Navarra, 31008 Pamplona, Spain; ageas@unav.es (A.G.); mcanela@unav.es (M.R.-C.); etoledo@unav.es (E.T.); mbes@unav.es (M.B.-R.); 3 CIBER Fisiopatología de la Obesidad y Nutrición, Instituto de Salud Carlos III, 28029 Madrid, Spain; 4 IdiSNA, Navarra Institute for Health Research, 31008 Pamplona, Navarra, Spain; 5 Department of Clinical Sciences, School of Biomedical and Health Sciences, Universidad Europea de Madrid, Laureate International Universities, 28029 Madrid, Spain; jjbeunza@unav.es (J.-J.B.)

**Keywords:** alcohol, Mediterranean drinking pattern, cardiovascular disease, cardiovascular mortality, cohort studies

## Abstract

Background: We assessed the still unclear effect of the overall alcohol-drinking pattern, beyond the amount of alcohol consumed, on the incidence of cardiovascular clinical disease (CVD). Methods: We followed 14,651 participants during up to 14 years. We built a score assessing simultaneously seven dimensions of alcohol consumption to capture the conformity to a traditional Mediterranean alcohol-drinking pattern (MADP). It positively scored moderate alcohol intake, alcohol intake spread out over the week, low spirit consumption, preference for wine, red wine consumption, wine consumed during meals and avoidance of binge drinking. Results: During 142,177 person-years of follow-up, 127 incident cases of CVD (myocardial infarction, stroke or cardiovascular mortality) were identified. Compared with the category of better conformity with the MADP, the low-adherence group exhibited a non-significantly higher risk (HR) of total CVD ((95% CI) = 1.55 (0.58–4.16)). This direct association with a departure from the traditional MADP was even stronger for cardiovascular mortality (HR (95% CI) = 3.35 (0.77–14.5)). Nevertheless, all these associations were statistically non-significant. Conclusion: Better conformity with the MADP seemed to be associated with lower cardiovascular risk in most point estimates; however, no significant results were found and more powered studies are needed to clarify the role of the MADP on CVD.

## 1. Introduction

Cardiovascular diseases (CVD) are the largest cause of mortality in the world and in the next years an increase in mortality due to CVD is expected, especially from ischemic heart disease and stroke [1]. However, some of the main risk factors for CVD are modifiable, such as diet and alcohol consumption.

Excessive alcohol intake is associated with several cardiac diseases and heart failure [2]. However, a J-shaped association between alcohol intake and the risk of cardiovascular diseases and cardiovascular mortality has been described [3,4]. Nevertheless, there are important dimensions of alcohol consumption that are associated with CVD [5]. Hereby an alcohol-drinking pattern can improve the assessment of risk of cardiovascular diseases, taking into account all the different aspects of alcohol intake.

The Mediterranean diet has proven to be a protective factor against cardiovascular mortality, myocardial infarction or stroke [6,7]. Moderate alcohol intake is an important component of this dietary pattern [8]. The Mediterranean alcohol-drinking pattern is characterized by red wine consumption, moderate intake, and wine consumed with meals and without excess [7,9,10]. On the other hand, binge-drinking and heavy irregular drinking patterns are associated with a higher cardiovascular risk [11].

Alcohol intake following a Mediterranean alcohol-drinking pattern (MADP) has shown a reduction of all-cause mortality compared with abstinence or poor adherence to this drinking pattern [12]. We aimed to prospectively assess the relationship between conformity to the MADP and the incidence cardiovascular events.

## 2. Methods

### 2.1. Study Population

The Seguimiento Universidad de Navarra (University of Navarra follow-up) (SUN) project is a prospective, multipurpose and dynamic cohort of university graduates in Spain. Enrolment is permanently open and follow-up is performed by mailed questionnaires every two years. A more detailed description of the methods can be found elsewhere [13]. The study protocol was approved by the Institutional Review Board of the University of Navarra.

**Figure 1:**
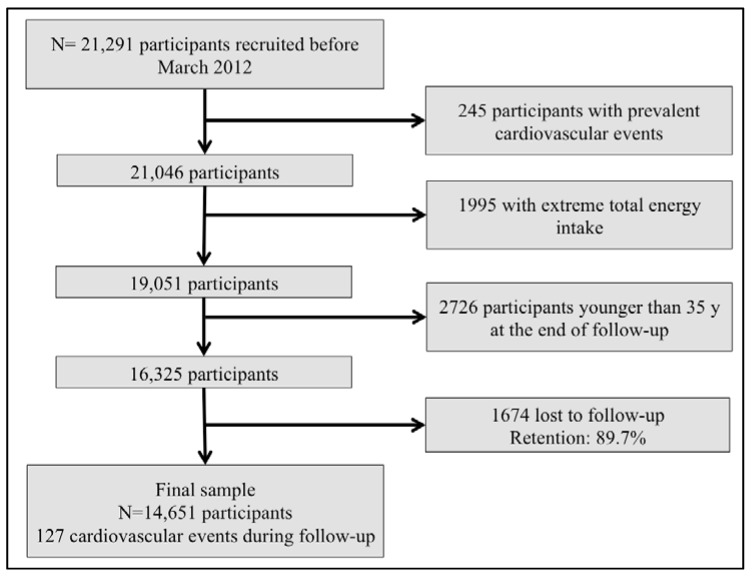
Flow chart of participants. The Seguimiento Universidad de Navarra (SUN) project (1999–2014).

We assessed 21,291 participants recruited before March 2012, to ensure they completed at least the 2-years follow-up questionnaire. We excluded 245 participants with baseline prevalent cardiovascular events, 1995 participants with total daily energy intake outside of predefined limits (<800 or >4000 Kcal/day among men, and <500 or >3500 Kcal/day among women) [14], and 2726 participants younger than 35 years old who were considered too young to have an alcohol-related cardiovascular event during follow-up. Out of the rest of participants, 1674 were lost to follow-up (retention in the cohort: 89.7%), leading to a final sample of 14,651 participants (Figure 1).

### 2.2. Exposure: Mediterranean Alcohol-Drinking Pattern

A 0-to-9-points score was used to capture the conformity to the traditional MADP [12]. The score was the sum of 7 items: (1) moderate total alcohol intake (2 points if the consumption was 10–50 g/day in men or 5–25 g/day in women; 1 point if the alcohol intake was lower than this range; 0 points if alcohol intake was higher than this range); (2) alcohol intake spread out over the week (we calculated the ratio between the number of drinking days per week and grams of alcohol intake during a week and categorised it in quartiles; 2 points were assigned to participants in the highest quartile, 1 point to participants in second and third quartiles and 0 points in the lowest quartile); (3) preference for wine (1 point if more than 75% of total alcohol consumed was from wine); (4) low consumption of spirits (1 point if spirits were equal or less than 25% of total alcohol intake); (5) preference for red wine over other types of wines (1 point if 75% or more of total wine intake was red wine); (6) avoidance of excess drinking occasions (1 point if the maximum number of drinks consumed in a single occasion did not exceed five drinks); and (7) consuming wine preferably during meals (1 point if 75% of wine or more was consumed with meals). Information about different alcoholic beverages consumption was assessed with a validated 136-item food-frequency questionnaire at baseline [15]. Other information about alcohol drinking habits was also inquired in the baseline questionnaire.

After the seven items were summed up, the score ranged from 0 to 9 points. We grouped participants into three categories according to their score: low adherence (0 to 2 points), moderate adherence (3 to 6 points) and high adherence (7 to 9 points). Abstainers were separately represented in a fourth group.

### 2.3. Outcome: Cardiovascular Events

The incident cardiovascular events were assessed as a combined outcome composed by incident fatal or non-fatal acute myocardial infarction with or without ST elevation, fatal or non-fatal stroke, and death due to other cardiovascular causes.

When participants reported one of these diagnostics in a follow-up questionnaire, they were asked for their medical reports, which were checked by a team of the study physicians blinded to nutritional information. Myocardial infarction was adjudicated using the universal criteria for myocardial infarction and non-fatal stroke was defined as a sudden onset focal-neurological lack with a vascular mechanism that lasted more than 24 h. Events were classified according with the International Classification of Diseases (ICD-10). We considered I21 and I63 codes to define cardiovascular events [16]. Deaths were reported to our research team by participant’s next of kin, work associates and postal authorities. The National Death Index was periodically checked to confirm the vital status of all our participants lost during follow-up.

### 2.4. Covariates Assessment

Information about anthropometric characteristics [17], classical cardiovascular risk factors (hypercholesterolemia, diabetes, hypertension [18]), metabolic syndrome [19], lifestyles (physical activity [20], sedentary activities, smoking status), use of cardiovascular-related medications, and prevalent stable coronary artery diseases was collected at baseline. We also evaluated the adherence to the Mediterranean dietary pattern, based on the food-frequency questionnaire information and using a classical score [21] but subtracting alcohol information out of this score to avoid overlapping with the main exposure.

### 2.5. Statistical Analysis

We fitted Cox regression models to estimate the relationship between adherence to the MADP and the risk of cardiovascular events or cardiovascular mortality. We estimated Hazard ratios (HR) and their 95% confidence intervals (CI) for abstainers, and for the groups of low and moderate-adherence, compared to the high-adherence group (reference category). In those models, exit time was defined as date of death, date of cardiovascular event or date of the last follow-up questionnaire, whichever came first, and age was used as the underlying time variable. We fitted an age and sex-adjusted model and a multiple-adjusted model. The multiple-adjusted model included as covariates the following potential confounders: body mass index (kg/m^2^), total energy intake (Kcal/day), smoking habit, physical activity (metabolic equivalent task-h/week), time spent watching television (h/day), cardiovascular treatment at baseline, and prevalent hypertension, hypercholesterolemia, hypertriglyceridemia, diabetes, cancer and stable coronary artery diseases. Moreover we assessed the interaction between the drinking pattern (MADP) and the Mediterranean dietary pattern on the risk of CVD or cardiovascular mortality using a likelihood ratio test in the fully-adjusted model to assess the interaction product-term. In addition, we assessed the individual contribution of every single component of the MADP score to account for the association with the incidence of cardiovascular events and cardiovascular mortality, adjusted for the effect of the rest of items in the MADP. For this aim, we fitted the same above-mentioned multiple-adjusted model but using the 7 items as exposure variables instead of the MADP. Analyses were performed using STATA version 12.0 StataCorp LP (College Station, TX, USA).

## 3. Results

A total of 127 cardiovascular events (55 myocardial infarction, 28 stroke and 44 cardiovascular deaths) were identified during follow-up (142,177 person-year; mean 9.7 years). Table 1 describes the main clinical, lifestyle and alcohol-related characteristics within categories of the MADP. Participants with higher adherence to the MADP were more likely to be men, older, and to have more classical cardiovascular risk factors (hypertension, hypercholesterolemia or diabetes) than the groups with lower adherence.

**Table 1:** Baseline characteristics of participants (mean (standard deviation) or %) according to adherence to the Mediterranean alcohol-drinking pattern. The Seguimiento Universidad de Navarra (University of Navarra follow-up) (SUN) project (1999–2014).

	Abstainers	Mediterranean Alcohol-Drinking Pattern		
		Low (0–2)	Moderate (3–6)	High (7–9)
*N*	2478	840	8192	3141
Age (years)	39.7 (11.1)	33.4 (9.0)	40.1 (11.0)	46.5 (10.8)
Sex (women %)	79.3	60.2	52.8	47.9
BMI (kg/m^2^)	23.2 (3.6)	23.5 (3.8)	23.9 (3.5)	24.2(3.3)
Current smokers (%)	14.7	32.1	24.1	16.9
Former smokers (%)	21.9	23.3	32.4	39.9
Physical activity (MET-h/week)	19.8 (24.1)	19.8 (21.0)	21.3 (22.0)	21.5 (21.0)
Time spent watching television (h/day)	1.6 (1.2)	1.9 (1.4)	1.6 (1.1)	1.5 (1.1)
Total energy intake (Kcal/day)	2284 (622)	2371 (621)	2360 (618)	2342 (613)
Prevalent hypertension (%)	8.4	6.4	8.8	13.1
Prevalent hypercholesterolemia (%)	16.4	14.4	18.0	25.3
Prevalent type 2 diabetes mellitus (%)	1.9	1.9	1.8	2.9
Prevalent or previous cancer (%)	4.8	2.4	3.8	5.2
Prevalent stable coronary artery diseases (%)	0.3	0.5	0.5	0.9
Cardiovascular-related medication use (%)	3.9	2.1	4.4	7.3
Mediterranean dietary pattern (0–8)	3.9 (1.8)	3.7 (1.6)	4.0 (1.7)	4.2 (1.7)
Alcohol intake (g/day)	-	7.4 (19.4)	9.4 (12.1)	11.8 (9.0)
Wine consumption (g/day)	-	1.6 (7.6)	3.9 (7.8)	9.1 (7.9)
Red wine consumption (g/day)	-	0.8 (4.3)	2.3 (5.7)	6.3 (7.2)
Spirit consumption (g/day)	-	3.3 (8.6)	1.8 (3.8)	0.5 (1.0)
>5 drinks in a single occasion (%)	-	62.9	36.9	9.0
Ratio (days/week): (g/week)	-	0.1 (0.1)	0.3 (0.4)	0.6 (0.6)
Proportion of wine consumed with meals	-	0.6 (6.8)	5.8 (27.6)	13.3 (36.0)

In the multivariable-adjusted model, a non-significant inverse association between adherence to the MADP and total cardiovascular events was observed. For the low MADP adherence group we found a HR (95% CI) of 1.55 (0.58–4.16), and for the moderate adherence group, a HR (95% CI) of 1.30 (0.85–1.98), both compared with the high adherence group (Table 2). A null association was observed among abstainers.

**Table 2:** Hazard ratios and 95% confidence intervals of incident cardiovascular events according to the categories of adherence to the Mediterranean alcohol-drinking pattern. The Seguimiento Universidad de Navarra (University of Navarra follow-up) (SUN) project (1999–2014).

	Abstainers	Mediterranean Alcohol Drink Pattern		
		Low (0–2)	Moderate (3–6)	High (7–9)
Cases/person-year	11/23,924	5/8681	73/79,695	38/29,877
Age and sex-adjusted model	0.88 (0.42–1.88)	2.25 (0.86–5.90)	1.37 (0.91–2.07)	1 (Ref.)
Multiple-adjusted model *	0.99 (0.46–2.12)	1.55 (0.58–4.16)	1.30 (0.85–1.98)	1 (Ref.)

* Adjusted for age, sex, Body Mass Index (kg/m^2^), total energy intake (Kcal/day), physical activity (MET-h/week), prevalent hypertension, prevalent hypercholesterolemia, prevalent hypertriglyceridemia, prevalent diabetes, prevalent cancer smoking habit (current smoker, former smoker or never smoker), Mediterranean dietary pattern (3 categories), watching television (h/day), cardiovascular treatment and prevalent stable coronary artery diseases. Ref.: Reference category.

When considering only cardiovascular mortality as outcome (Table 3), the point estimates were stronger for all categories, but they remained non-significant. Compared to high adherence, the category of low adherence to the MADP showed a HR (95% CI) of 3.35 (0.77–14.5). For the comparison between the category of moderate adherence *versus* high adherence the association was statistically significant (multivariable-adjusted HR (95% CI) = 2.64 (1.11–6.23)). And for abstainers *versus* high adherence, we found a non-significant direct association (HR (95% CI) = 1.90 (0.52–6.98)).

**Table 3:** Hazard ratios and 95% confidence intervals of cardiovascular mortality according to the categories of adherence to the Mediterranean alcohol-drinking pattern. The Seguimiento Universidad de Navarra (University of Navarra follow-up) (SUN) project (1999–2014).

	Abstainers	Mediterranean Alcohol Drink Pattern		
		Low (0–2)	Moderate (3–6)	High (7–9)
Cases/person-year	5/23,891	3/8673	27/79,449	9/29,719
Age and sex-adjusted model	1.48 (0.42–5.23)	5.94 (1.48–23.9)	2.67 (1.19–5.98)	1 (Ref.)
Multiple-adjusted model *	1.91 (0.52–6.98)	3.35 (0.77–14.5)	2.64 (1.11–6.23)	1 (Ref.)

* Adjusted for age, sex, Body Mass Index (kg/m^2^), total energy intake (Kcal/day), physical activity (MET-h/week), prevalent hypertension, prevalent hypercholesterolemia, prevalent hypertriglyceridemia, prevalent diabetes, prevalent cancer smoking habit (current smoker, former smoker or never smoker), Mediterranean dietary pattern (3 categories), watching television (h/day), cardiovascular treatment and prevalent stable coronary artery diseases. Ref.: Reference category.

In our sample, among drinkers, the MADP showed a correlation of small magnitude with the adherence to the Mediterranean dietary pattern (excluding alcohol). The Spearman’s correlation coefficient was 0.095. Moreover, we explored the interaction between MADP and Mediterranean dietary pattern. There was no statistically significant interaction on CVD (*p* for interaction = 0.86) nor on cardiovascular mortality (*p* for interaction = 0.83).

**Figure 2:**
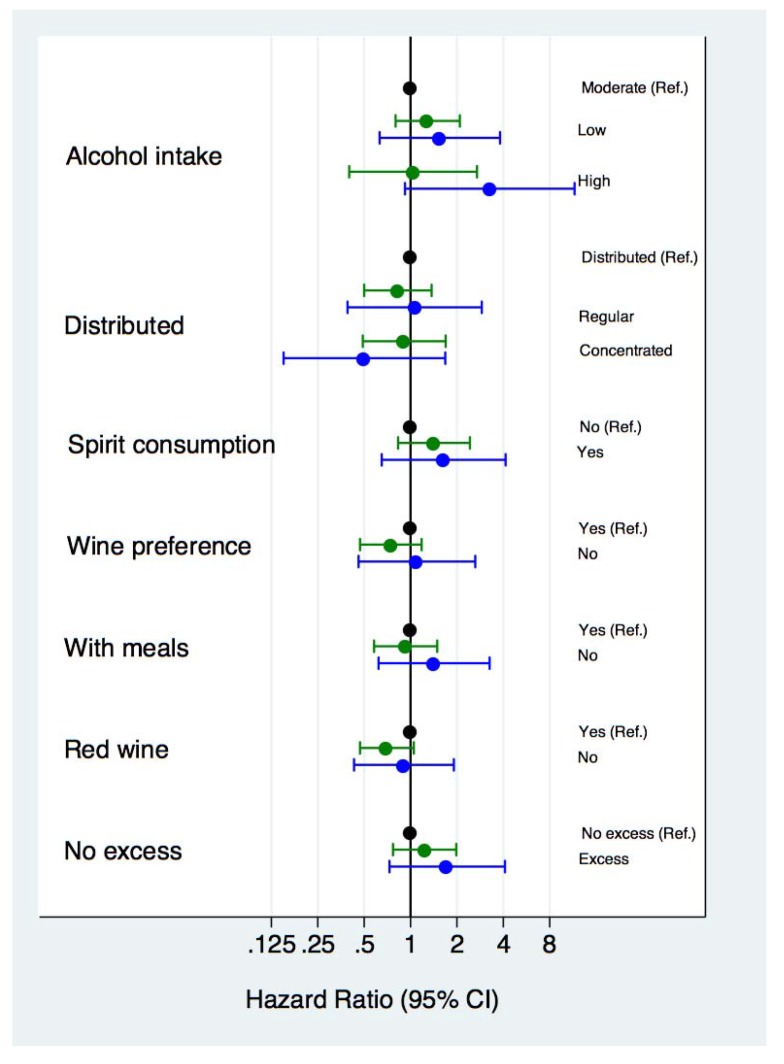
Cardiovascular disease (green) and cardiovascular mortality (blue) hazard ratios (HR) and 95% confidence intervals for each component of the Mediterranean alcohol-drinking pattern.

In Figure 2 we show the association between each component of MADP and cardiovascular events or cardiovascular mortality. Moderate alcohol intake, low spirit consumption and avoidance of binge drinking (“no excess” in Figure 2) were the items of the MADP score associated with the lowest risks for CVD events or mortality although all these associations were statistically non-significant. However, we observed a significant higher risk of cardiovascular events (HR (95% CI) = 1.71 (1.05–2.78), *p* = 0.03) and cardiovascular mortality (HR (95% CI) = 3.05 (1.10–8.44), *p* = 0.03) in participants who did not meet these three characteristics of the MADP compared with those participants who did meet them.

## 4. Discussion

In the present study, the traditional Mediterranean alcohol-drinking pattern was inversely but non-significantly, associated with the incidence of cardiovascular disease and cardiovascular mortality. In that pattern, seven alcohol-related characteristics were considered altogether.

Although light to moderate alcohol intake is acknowledged to be associated with reduced risk of cardiovascular outcomes [22], even in individuals with low cardiovascular risk [23], there are other dimensions of alcohol intake that are important. Numerous studies have investigated the relationship between alcohol intake and cardiovascular events [3], but a fewer number of studies have assessed the drinking pattern [24]. Although some studies have investigated the role of alcohol intake in relation to health in Mediterranean populations and in the context of a Mediterranean diet [25,26], to our knowledge, this is the first study that evaluates the overall drinking pattern jointly taking into account several different dimensions of alcohol intake integrated in a combined score. This is important because the effects previously found for single dimensions of alcohol drinking patterns may be confounded by the other dimensions that were not introduced in the model.

Better adherence to the Mediterranean alcohol-drinking pattern was associated with a lower total mortality compared with poorer adherence or abstention [12]. Our results point in the same direction of a healthy effect of this pattern, although the results for CVD that we report here were statistically non-significant. Results might be different due to a stronger effect and a higher number of cases in the mortality analyses than in CVD analyses.

Additionally, the observed association was stronger for cardiovascular mortality than for non-fatal cardiovascular events, though both were non-significant. A possible explanation may be a differential effect of the alcohol-drinking pattern on the progression of cardiovascular events and on the case-fatality rate. For instance, wine increases the expression of endothelial nitric oxide synthase [27] and inhibits platelet activity and thrombosis [28] that contribute to improve coronary flow [29]. These effects can be useful to prevent lethal events. Another possible interpretation is that participants with low-adherence are less concerned about their health status, might be less likely to look for medical care when they exhibit early clinical symptoms or do not comply with the medical advices when the situation is irreversible. However, our analyses were adjusted for a wide arrange of potential confounders that probably balanced participants in these aspects.

Individually, we did not find any independent statistically significant association for any of the seven items of the MADP, adjusting for the rest of items. However, moderate alcohol intake (10–50 g/day in men or 5–25 g/day in women), low spirits consumption (less than 25%) and non-excessive consumption in a single day (never more than five drinks/occasion) were strongly, though non-significantly, associated with CVD or cardiovascular mortality. Our results regarding moderate alcohol intake are consistent with previous studies [3,4]. Additionally, episodes of large amounts of alcohol intake in a single day (*i.e.*, binge drinking) have been associated with a high risk of coronary heart disease and mortality in several studies [5,30]. Moreover, alcohol intake distributed over the week has been inversely associated with the risk of myocardial infarction [24] independently of the type of beverage or the proportion consumed with meals.

On the other hand, the importance of the other items included in the MADP is still controversial. There is some evidence to support the benefits of wine on cardiovascular outcomes and mortality [31,32], but some studies have found no differences between the different types of wine [33], or different types of alcoholic beverages. Otherwise, red wine consumption has showed to be associated with increased levels of high-density lipoprotein (HDL) cholesterol, apolipoprotein A1 and adiponectin; a decrease in fibrinogen concentrations and beneficial effects on inflammation and inhibition of platelet aggregation [34,35]. Red wine polyphenol extracts have cardioprotective properties, including antioxidant properties, which have been repeatedly assessed [36]. Moreover, consumption of red wine with meals exhibited further beneficial metabolic effects in experimental studies [37,38].

However, moderate wine-drinkers usually have healthier lifestyles than others types of alcohol drinkers: less smoking and more physical activity [39], increased fruit and vegetables consumption and low red and fried meat consumption [40]. Otherwise, heavy and binge drinkers use to present unhealthier behaviors, such as poor-quality diets, low physical activity, and a general tendency to reckless actions that determine an increased risk of mortality [41,42]. Some authors claim that the difference in health outcomes between different drink-preferences or drinking patterns is due to confounding by these differences in lifestyles. Nonetheless, we adjusted our models for several potential confounding factors.

We must acknowledge some limitations in our study. First, our analyses may lack statistical power since our cohort is relatively young and few incident cases of CVD were observed during follow-up. We estimated statistical power using the Stata 12.0 command for a Cox proportional hazards model (stpower cox) under the following assumptions: expected hazard ratio = 2; expected absolute risk of CVD = 2%; expected standard deviation of the exposure variable = 0.5; total sample size *n* = 3800 (we assumed a comparison only between extreme categories of alcohol drinking pattern); and a 2-tailed alpha risk = 0.05. Under these assumptions the estimated statistical power was 85.6%. These assumptions were set a priori. Methodologists [43] have pointed out that it would be futile to estimate power using as assumptions the observed results, because it “tautologically yields an answer of low power”. They proposed to use instead the width of the confidence interval to appraise the statistical power because “The power of the trial is expressed in that confidence interval”.

Second, our cohort does not constitute a representative sample of the general population and therefore generalization of the results must be based on biological mechanisms instead of statistical representativeness. Third, both exposure and part of the outcome information were self-reported, and some degree of misclassification is possible. In studies about alcohol intake, under-reporting is always a possibility. However, we believe that since alcohol was assessed together with many other dietary intakes, and since participants in our sample are highly educated and health conscious, the degree of under-reporting was not of important magnitude in our study. Nevertheless, if there was some degree of under-reporting, we would expect it to lead to a non-differential misclassification and, hence the most likely bias would be towards the null value. And finally, although we adjusted our models for a wide array of potential confounders, residual confounding is still possible.

On the other hand, our study has several important strengths. The sample size is relatively large, the follow-up is long, and the retention in the cohort is high. Volunteers participating in this cohort are highly educated subjects and more than half of them are health professionals themselves; although the presence of selection bias is possible, these facts reduce the potential of confounding by educational levels and leads to better quality in self-reported data, improving thus the internal validity of the study. Moreover, alcohol intake was collected with a previously validated food-frequency questionnaire [15], and in the validation study, the correlation coefficient for alcohol intake (*r* = 0.88) was higher than that for most other nutrients. Finally, outcomes were confirmed by checking the medical records of participants, leading to a lower degree of outcome misclassification.

## 5. Conclusions

Though non-significantly, the MADP seemed to be associated with reduced incidence of cardiovascular events and cardiovascular mortality. Moderate alcohol intake, low spirits consumption and avoidance of binge drinking, were the three items with the strongest association. However, no significant results were found and more powered studies are needed to clarify the role of the MADP on cardiovascular disease and cardiovascular mortality, using the overall drinking pattern approach to evaluate the different aspects of alcohol intake altogether.
